# Posttraumatic Stress Disorder Is Associated With Emotional Eating

**DOI:** 10.1002/jts.21824

**Published:** 2013-07-25

**Authors:** Lisa S. Talbot, Shira Maguen, Elissa S. Epel, Thomas J. Metzler, Thomas C. Neylan

**Affiliations:** 1 San Francisco VA Medical Center, San Francisco, California, USA; 2 Department of Psychiatry, University of California, San Francisco, California, USA

## Abstract

The present study investigated the relationship between posttraumatic stress disorder (PTSD) and emotional eating in a sample of medically healthy and medication-free adults. Participants with PTSD (*n* = 44) and control participants free of lifetime psychiatric history (*n* = 49) completed a measure of emotional eating. Emotional eating is the tendency to eat or overeat in response to negative emotions. PTSD participants exhibited greater emotional eating than control participants (η^2^ = .20) and emotional eating increased with higher PTSD symptom severity (*R*^2^ = .11). Results supported the stress-eating-obesity model whereby emotional eating is a maladaptive response to stressors. Over time, this could lead to weight gain, particularly abdominal stores, and contribute to higher risk for comorbid medical disorders. Findings suggest the importance of future longitudinal research to understand whether emotional eating contributes to the high rates of obesity, diabetes, and heart disease in PTSD.

Accruing evidence suggests that posttraumatic stress disorder (PTSD) is associated with increased risk for obesity (Pagoto et al., 2012; Scott et al., 2008) and other related medical comorbidities including diabetes and heart disease (e.g., Kubzansky, Koenen, Spiro, Vokonas, & Sparrow, 2007). One possible explanation for the relationship between PTSD and obesity is eating in response to chronic stress. This tendency can be referred to as emotional eating or stress eating (e.g., Pinaquy, Chabrol, Simon, Louvet, & Barbe, 2003; Tomiyama, Dallman, & Epel, 2011). Indeed, a substantial literature suggests that both perceived stress and chronic stress exposure are associated with overeating (e.g., Adam & Epel, 2007; Dallman, 2010; Groesz et al., 2012; Warne, 2009). Over time, this behavior can lead to weight gain, and as such the process can be termed the stress-eating-obesity model.

Biological mechanisms may underpin this drive to eat in response to stress. Corticotrophin-releasing hormone (CRH) initiates the stress response by regulating the hypothalamus–pituitary–adrenal (HPA) axis. The resulting glucocorticoid secretion contributes to abdominal fat deposition directly (Bjorntorp & Rosmond, 2000; Dallman, Pecoraro, & la Fleur, 2005) as well as indirectly via increased intake of “comfort foods” (i.e., foods high in fat and/or sugar that individuals are more likely to eat when stressed; e.g., Zellner et al., 2006). Animal studies provide evidence that the comfort food (a component of emotional eating) and abdominal fat in turn dampen the HPA axis response (i.e., reduce hypothalamic CRH expression; e.g., Dallman, 2010; Foster et al., 2009). A recent human study also supported this model. Healthy women high in chronic stress reported greater emotional eating, higher abdominal fat, and lower cortisol response during a laboratory stressor compared to healthy women low in chronic stress (Tomiyama et al., 2011).

This psychobiological stress-eating-obesity model has relevance to PTSD, given that (a) PTSD is characterized by chronic stress; (b) PTSD is associated with obesity (Pagoto et al., 2012); and (c) some research suggests lower cortisol levels in PTSD (for a review, see Yehuda, 2006). Given these premises, the present study sought to test an early component of this model in PTSD: emotional eating. Specifically, we examined whether PTSD is associated with self-reported emotional eating in a young, healthy, unmedicated sample. First, we predicted that relative to the control group, the PTSD group would exhibit higher emotional eating scores. Second, we predicted that emotional eating would correlate with PTSD severity.

## Method

### Participants

Participants included 93 adults recruited from newspaper advertisements, web-based postings, flyers in community-based outpatient clinics, and from the clinical PTSD program at a Veterans Affairs (VA) medical center. Participants were relatively young, healthy, and medication-free in order to examine PTSD without the potential confounds of physical illness, medication, and aging. The majority of participants were nonveteran. The sample included 44 individuals with current chronic PTSD (50% women) and 49 control participants without PTSD (55% women), ranging in age from 20 to 50 years.

Chronic PTSD was defined by PTSD criteria according to the *Diagnostic and Statistical Manual of Mental Disorders* (4th ed., text rev.; *DSM-IV-TR*; American Psychiatric Association, 2000) or by the Clinician-Administered PTSD Scale (CAPS; Blake et al., 1995) score of > 40 for at least 3 months. Only six participants with PTSD had a CAPS score of < 40; they did not differ on demographic or outcome variables compared to the individuals with a CAPS score of > 40. Control participants were negative for lifetime PTSD, had a CAPS score of < 20, and were free from lifetime major depressive disorder and panic disorder. Female participants were studied during the follicular phase of their menstrual cycle. Exclusion criteria for both groups included high body mass index (BMI; > 35); neurologic disorder or systemic illness affecting central nervous system function; pregnancy; use of psychiatric, anticonvulsant, antihypertensive, sympathomimetic, estrogen replacement therapy, or steroid medication; lifetime history of any psychiatric disorder with psychotic features; bipolar disorder; obsessive–compulsive disorder; alcohol abuse or dependence within the past 2 years; and substance abuse or dependence in the past year.

### Measures

#### Clinician-Administered PTSD Scale.

Current PTSD was assessed with the CAPS (Blake et al., 1995). The CAPS has excellent test-retest reliability (*r* = .92 to .99) and internal consistency (α = .80 to .90; Weathers, Keane, & Davidson, 2001).

#### Structured Clinical Interview for DSM-IV (SCID).

Diagnoses other than PTSD were assessed with the SCID (see First, Spitzer, Williams, & Gibbon, 1996). The SCID has been shown to have good reliability (e.g., Williams et al., 1992).

All diagnoses were made by trained clinical interviewers who calibrated their assessments at weekly case consensus meetings, supervised by an experienced doctoral-level clinical psychologist.

#### Dutch Eating Behavior Questionnaire.

The 13-item emotional eating subscale from the Dutch Eating Behavior Questionnaire (DEBQ; Van Strien, Frijters, Bergers, & Defares, 1986) was used to measure emotional eating. Items are rated on a 5-point Likert scale, ranging from 1 = *never* to 5 = *very often*. Higher scores on the total emotional eating subscale score reflect more emotional eating. The emotional eating subscale displays good internal consistency (α = .95 in the present sample) and factorial validity (e.g., Van Strien et al., 2007) and good validity for food consumption (Van Strien & Van der Laar, 2008).

#### Body Mass Index.

BMI was calculated from height and weight measurements obtained by trained clinical research staff at a University of California, San Francisco Hospital.

### Procedure

All research was approved by the Committee on Human Research at the University of California, San Francisco and at the San Francisco Veterans Affairs Medical Center. Participants who were likely to be eligible after a telephone screen visited the laboratory for administration of the CAPS and SCID. Eligible participants then completed the DEBQ. Approximately 1 week later, participants visited the hospital for height and weight measurements.

## Results

### Participant Characteristics

There were no significant differences between groups in age, gender, or years of education. The PTSD group had fewer Caucasians, more individuals who were divorced or separated, a higher BMI, and more veterans (see Table 1). The average CAPS score for the PTSD group was 54.55 (*SD* = 15.74) and the average CAPS score for control participants who experienced a *DSM-IV* Criterion A event (*n* = 11) was 0. Eighteen percent of PTSD participants (*n* = 8) met criteria for a current major depressive episode.

### Emotional Eating

A univariate analysis of covariance was conducted on the emotional eating score with group (PTSD, control) as the between-subjects variable. BMI, race, marital status, and veteran status were included as covariates given the baseline group differences. Gender and depression status were also included as covariates. There was a main effect of group, *F*(1, 80) = 20.21, η^2^ = .20, *p* < .001, with the PTSD participants exhibiting more emotional eating than control participants^1^ (PTSD: *M* = 31.27, *SD* = 11.11; control: *M* = 23.61, *SD* = 8.64). There was a significant main effect of marital status, *F*(3, 80) = 4.72, η^2^ = .15, *p* = .004. There was no difference in emotional eating in the control group for married compared to unmarried (*M* = 24.71, *SD* = 13.59, and *M*= 23.42, *SD* = 7.75, respectively), but the married individuals with PTSD had significantly lower emotional eating scores than the unmarried individuals (*M* = 15.50, *SD* = 3.54, and *M*= 32.02, *SD* = 10.79, respectively). There were no other significant main effects. There were no significant interactions of Group × Marital Status or Group × Gender.

### PTSD Severity and Emotional Eating

**Figure 1.**
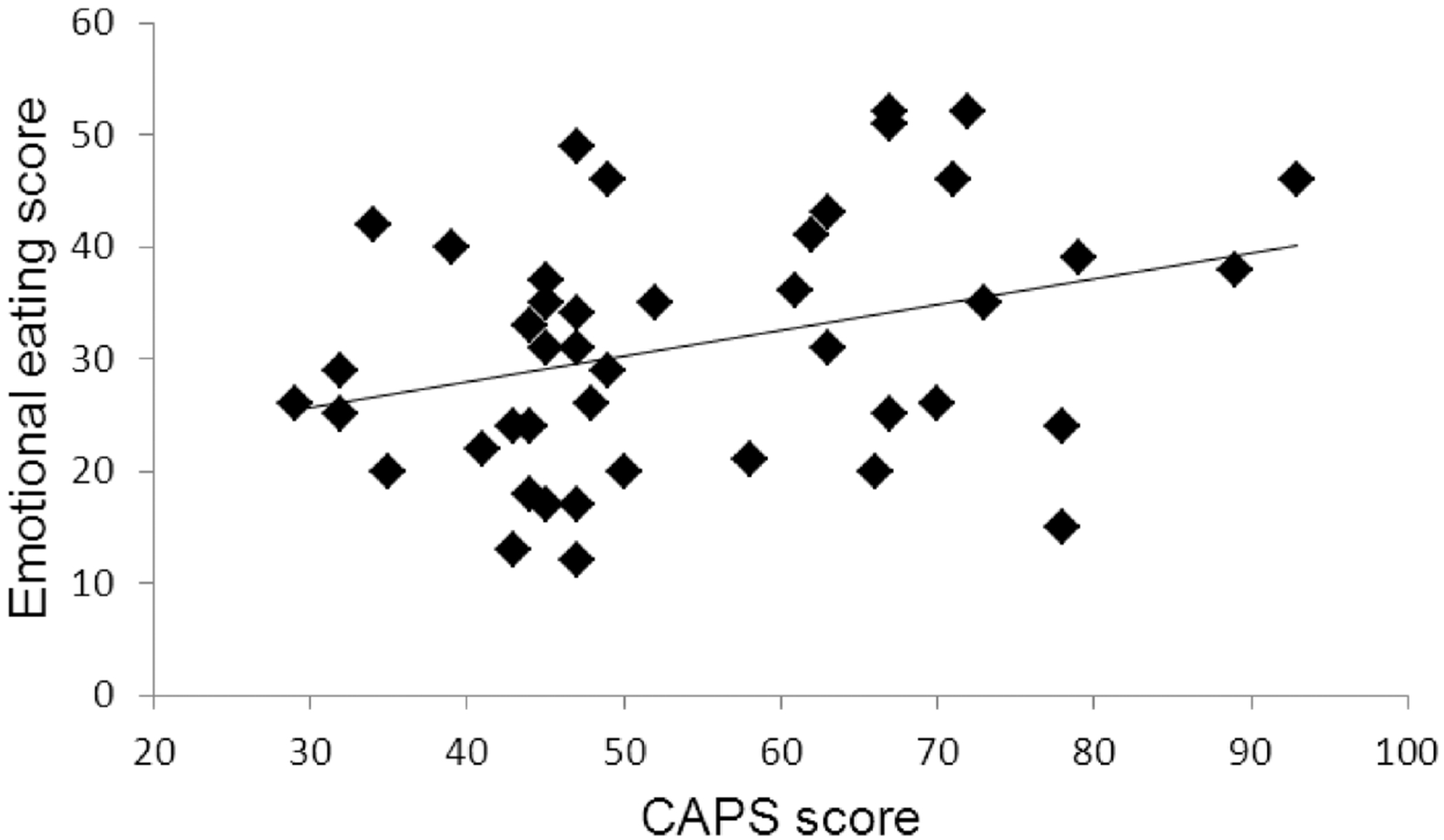
Scatterplot demonstrating the relationship between Clinician-Administered PTSD Scale (CAPS) score and emotional eating score in the posttraumatic stress disorder (PTSD) group. Plotted line represents the fit line.

To assess whether PTSD severity is associated with emotional eating score, a linear regression was conducted. When the emotional eating score was regressed on CAPS score in the PTSD group, more severe PTSD was associated with increased emotional eating, β = .33, *SE* = .10, *t*(42) = 3.20, *p* = .031 (Figure 1). PTSD severity also explained a significant proportion of variance in eating behavior, *R*^2^ = .11, *F*_change_(1, 42) = 5.01, *p* = .031.

## Discussion

Individuals with PTSD exhibited more emotional eating than individuals in the healthy control group. The preliminary data suggest that it may be an important topic for further research to determine whether emotional eating contributes to the high rates of obesity and related medical problems in PTSD. A recent study observed that returning veterans—a group with a high rate of PTSD—experience a burst of weight gain following military discharge (Koepsell, Littman, & Forsberg, 2012); it is possible that emotional eating patterns associated with PTSD may in part explain such weight gain.

Interestingly, in the present study the PTSD group demonstrated more emotional eating even after controlling for BMI, gender, and depression, none of which exhibited effects on emotional eating. There was, however, an effect of marital status on emotional eating. Unmarried individuals with PTSD reported more emotional eating than married individuals. It is difficult to draw conclusions, however, given that there were only two married participants with PTSD in the sample. Nonetheless, it will be important to consider psychosocial factors such as marital status in future research, particularly given that the relationship between emotional eating and social support is unclear (Raspopow, Matheson, Abizaid, & Anisman, 2013). We also observed that more severe PTSD as measured by the CAPS was associated with more emotional eating, consistent with recent research indicating that more psychological stress is associated with greater emotional eating (Tomiyama et al., 2011).

Several limitations are important to consider. First, the key construct of emotional eating was measured by self-report. Although the DEBQ has good validity for food consumption, future studies could use a multimethod approach including labbased observations. Second, the cross-sectional nature of the study limits conclusions about the potential relevance of the stress-eating-obesity model in PTSD. Future research should include longitudinal assessments. Third, the relatively small sample size may have reduced the ability to detect group differences and the influence of other variables (e.g., depression). Moreover, because the groups were not randomized, it is possible that group differences could be related to potential unmeasured confounds. Finally, the relatively young, healthy, low BMI, medication-free sample is not representative of the general PTSD population. Overall, the limitations inherent to this study suggest that the results are preliminary. Nonetheless, the initial evidence for emotional eating in PTSD indicates a need for future longitudinal research to assess whether emotional eating serves as an introductory pathway from stress to diseases such as obesity, diabetes, and heart disease in PTSD.

## Figures and Tables

**Table 1:** Characteristics of Healthy Medication-Free PTSD Participants and Age-Matched Controls

	Group				
	PTSD group (*n* = 44)		Control group (*n* = 49)		
Variable	*M*	*SD* or %	*M*	*SD* or %	Test statistic
Age (*SD*)	30.55	6.57	30.04	7.91	*t*(91) = .33
Gender (%)					χ^2^(1) =.24
Male	22	50	22	45	
Female	22	50	27	55	
Years of education (*SD*)	14.86	2.20	15.36	2.04	*t*(91) = 1.12
Race (%)					χ^2^(4) = 11.05*
African American	5	11	1	2	
Asian American	3	7	7	14	
Caucasian	27	61	39	80	
Other	7	16	1	2	
Unknown	2	5	1	2	
Marital Status (%)					χ^2^(3) = 8.86*
Single	34	77	41	84	
Married/Partnered	2	5	7	14	
Divorced	5	11	1	2	
Separated	3	7	0	0	
Veteran status					χ^2^(1) = 12.45***
Civilian	34	77	49	100	
Veteran	10	23	0	0	
Body mass index	26.95	4.64	24.40	3.78	*t*(90) = −2.99**
CAPS	54.55	15.74	0.00	0	*t*(43) = −22.99***
Current depression					χ^2^(1) = 9.75**
Absent	36	82	49	100	
Present	8	18	0	0	

*Note*. PTSD = posttraumatic stress disorder; CAPS = Clinician-Administered PTSD Scale. Three participants who described their race as Hispanic comprised the participants in the ‘Unknown’ race category.

* *p* < .05.

** *p* < .01.

*** *p* < .001.

## Footnotes

1 A subset of participants (PTSD, *n* = 11, 25%; control, *n* = 20, 41%) were given a questionnaire with a missing item (#10). We note a correlation of *r* = .995, *p* < .001, between total emotional eating score in the sample who completed the questionnaire with all items included and those who completed it without item #10. To be more conservative, the analyses presented in this article were conducted with item #10 excluded for all participants.
